# Petrified Human Body

**Published:** 1846-01-01

**Authors:** 


*Petrified Human Body.*—Some months since an account of the petrified body of a female buried many years ago near Montreal, Canada, circulated through the newspapers of the country. The same body (as we presume) has lately been imported into the United States for exhibition. It was carried to Boston, and there exhibited, as we learn from the Boston Medical and Surgical Journal, "tightly screwed up in a box," so that the curious observer was debarred from touching it, but presenting, as seen through glass, the appearance of solid stone. The British American Journal of Montreal, in the No. for November, explains the precautions against close inspection of it. The editor had an opportunity of examining it, and of removing a portion of the fleshy part of the arm, which proved to be a beautiful specimen of *adipocire*. As such it would be an interesting *subject* for examination, but to pass it off for a petrifaction is a contrivance of cupidity, which the British Journal very properly exposes.

